# Fabrication of Core-Shell Chopped C_f_-Phenolic Resin Composite Powder for Laser Additive Manufacturing of C_f_/SiC Composites

**DOI:** 10.3390/polym13030463

**Published:** 2021-02-01

**Authors:** Xiao Chen, Jie Yin, Xuejian Liu, Aidong Xia, Zhengren Huang

**Affiliations:** 1 State Key Laboratory of High Performance Ceramics and Superfine Microstructures, Shanghai Institute of Ceramics, Chinese Academy of Sciences, Shanghai 200050, China; chenxiao@student.sic.ac.cn (X.C.); xiaaidong@student.sic.ac.cn (A.X.); 2 College of Materials Science and Opto-Electronic Technology, University of Chinese Academy of Sciences, Beijing 100049, China; 3 Ningbo Institute of Materials Technology and Engineering, Chinese Academy of Sciences, Ningbo 315201, China

**Keywords:** chopped C_f_, composite powder, silane coupling agent, laser additive manufacturing

## Abstract

Laser additive manufacturing is a promising technique for the preparation of complex-shaped SiC composites. High-quality powders are critical for high-precision laser printing. In this work, core-shell C_f_ @phenolic resin (PR) composites for selective laser sintering of carbon fiber reinforced silicon carbide (C_f_/SiC) composites were fabricated by surface modification using 3-aminopropyltriethoxy silane coupling agent (KH550) in combination with planetary ball milling. PR coated uniformly on the fiber surface to form a core-shell structure. The effects of PR on the morphology, elemental composition, interfacial interactions, and laser absorption of the core-shell composite powder were investigated in detail. Results indicated that the composite powder exhibited good laser absorption within the infrared band.

## 1. Introduction

Silicon carbide (SiC) is a strong covalent compound composed of CSi_4_ and SiC_4_ tetrahedron lattices interpenetrating each other, which has been widely applied in aerospace, nuclear, and optical fields ascribed to its incomparable strength, thermostability, and corrosion resistance [1,2,3,4,5]. Nevertheless, its intrinsic brittleness is a major obstacle for high-temperature structural applications [6]. Carbon fiber reinforced SiC (C_f_/SiC) composites exhibit lightweight and superior reliability that are promising candidates for structural applications. In comparison with continuous carbon fiber, chopped carbon fiber reinforced ceramic matrix composites have attracted more attention due to the fabrication flexibility [7].

The main methods for fabricating C_f_/SiC composites include the gas phase method (chemical vapor infiltration, CVI), the liquid methods (precursor impregnation and pyrolysis, PIP; liquid silicon infiltration, LSI), and the ceramic method (hot pressing sintering, HPS; spark plasma sintering, SPS) [8]. Huang’s team fabricated C_f_/SiC via slip casting combined with LSI with the flexural strength of 412 ± 47 MPa [9]. Jiang’s team fabricated C_f_/SiC composites by SPS. Their study indicated that the ultrarapid heating and cooling process during SPS resulted in thermal residual stress and the generation of cracks, yet the composites exhibited a non-catastrophic fracture due to the introduction of chopped carbon fibers [10]. Jiang’s team reported the C_f_/SiC composites prepared by HPS had superior mechanical properties. The flexural strength and fracture toughness were 395.5 MPa and 6.8 MPa m^1/2^, respectively [11]. However, it is rather difficult to fabricate complex-shaped components via the aforementioned methods.

Additive manufacturing (AM) or, namely, 3D printing provides flexibility to fabricate complex-shaped components [12,13]. Selective laser sintering (SLS) is a promising AM technology mainly because of its high accuracy, high throughput, and high efficiency [14,15]. SLS utilizes a laser generated by a stimulated emission combined with population inversion as an energy source to soften and bond materials [16]. Moreover, the excessive powder mass not only provides support for the built components but can also be recollected and recycled [17].

SLS can be divided into direct SLS and indirect SLS. Compared with direct SLS, indirect SLS shows great advantages including low thermal residual stress, fewer cracks, and high density and thus receives increasing attention on SiC ceramic and composites [18]. Zeng’s team reported the SiC ceramic prepared by indirect SLS and PIP exhibited excellent high-temperature strength (1600 °C, 203.7 MPa). In their study, the epoxy resin acted as a binder to bond SiC powders [19]. Yan’s team utilized a solvent evaporation method to fabricate phenolic resin coated carbon fiber and constructed a carbon fiber perform via indirect SLS. The final C_f_/SiC composites were obtained via polymer infiltration, pyrolysis, and LSI [20].

During the indirect SLS, the lower melting point polymer binder melts and bonds the raw material powder together ascribed to the thermal effect of the laser. Further, the binder is transformed into inorganic carbon upon firing. “Printable” powder is critical for indirect SLS. The requirements of high-quality powder suitable for SLS processing mainly include high laser absorption and low binder content. However, the modification of raw powders is normally required.

In this paper, a core-shell chopped C_f_ @phenolic resin composite powder with excellent laser absorption for indirect SLS was fabricated via a coupling agent grafting method combined with ball milling. A 3-aminopropyltriethoxy silane coupling agent (KH550) was grafted on the surface of the carbon fiber to improve the wettability and interfacial compatibility between the carbon fibers and the phenolic resin coating. Simultaneously, the surface elemental composition, chemical structure, as well as microcrystalline structure were investigated. Phenolic resin (PR) was used as a binder to form core-shell chopped C_f_ @phenolic resin composite powder for the subsequent SLS process. The effects of PR on the morphology and laser absorption of the composite powder were discussed.

## 2. Experimental

### 2.1. Materials

The carbon fiber was supplied by Shanghai Lishuo Composite Co., Ltd. (Shanghai, China). Table 1 shows the basic properties of carbon fiber. The 3-aminopropyltriethoxy silane coupling agent (KH550, Nanjing Chuangshi Chemical Co., Ltd.; Nanjing, China) was used as the surfactant. Thermoplastic phenolic resin was selected as the coating substance (Shaoxing Shangyu Ziqiang Polymer Materials Co., Ltd.; Shaoxing, China).

### 2.2. Experimental Process

**Figure 1:**
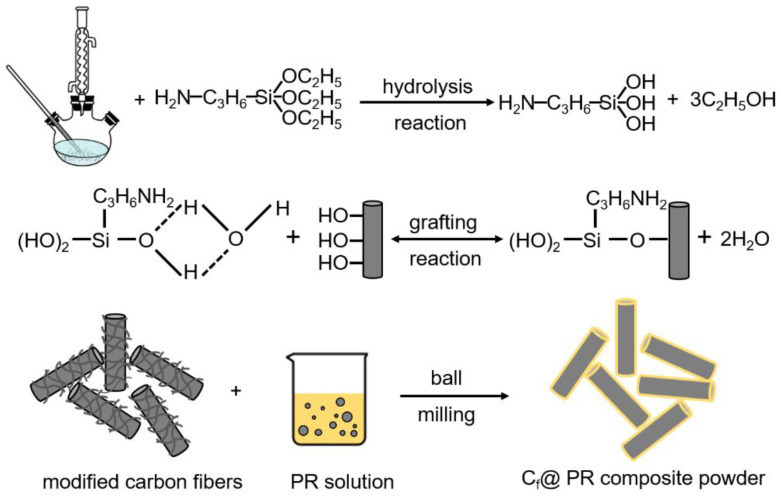
Procedures for fabrication of C_f_@ PR composite powder.

(1) Surface modification of carbon fiber: In this work, the carbon fiber was modified by 3-aminopropyltriethoxy silane coupling agent (KH550). The KH550 as a bifunctional compound can improve the wettability and compatibility between the inorganic carbon fiber core and organic phenolic resin shell [21]. Meanwhile, the presence of long-chain KH550 is beneficial for the PR to coat onto the surface of carbon fiber by the molecular chain entanglement mechanism [22]. An amount of KH550 was added into the mixed solvent composed of 30 wt% deionized water and 70 wt% absolute ethanol in a three-necked flask to react for 30 min in order to make the KH550 adequately hydrolyzed at 75 °C. Subsequently, the carbon fiber powder was added into the reactor under magnetic stirring at 75 °C for 4 h. Finally, the modified fibers were separated from the mixed solution and ultrasonically cleaned with alcohol three times to remove excessive KH550 [23]. The obtained fibers were dried in an oven at 70 °C for 12 h.

(2) Preparation of core-shell chopped C_f_ @phenolic resin composite powder: The phenolic resin was ultrasonically dispersed completely in ethanol. Then, the KH550 modified carbon fiber and the PR solution were transferred into a container and ball milled at 300 rpm for 1 h. The mass ratio of SiC balls, KH550 modified carbon fiber, and ethanol is 2:1:1. After ball milling, the suspension was separated and dried in an oven at 60 °C. Finally, the C_f_ @PR was obtained via grinding the dried sample with an agate mortar and sieving through a 100 mesh. In this work, the C_f_ @PR composite powders with a PR volume content of 10 vol%, 15 vol%, 20 vol%, 25 vol%, and 30 vol% were fabricated. Figure 1 shows the detail procedures for the fabrication of C_f_ @PR composite powders.

### 2.3. Characterizations

The infrared spectra of carbon fiber before and after modification by KH550 as well as C_f_ @PR were obtained via a Spotlight 400 Fourier Transform Infrared Spectrometer (FTIR) to analyze the surface functional groups and laser absorption from 4000 cm^−1^ to 400 cm^−1^ wavenumbers. The samples and dried KBr were ground in an agate mortar to uniformly mix them, and then dried under an infrared lamp. Finally, the samples were compressed and tested under ambient conditions. An X-ray photoelectron spectroscopy (XPS, ESCAlab250, Thermo Fisher Scientific, Waltham, MA, USA) with a monochromatic Al Kα X-ray source was used to characterize the surface elemental composition and atomic concentrations. A Raman spectroscopy was used to analyze the microstructure of carbon fiber before and after KH550 modification. The argon laser with 532 nm was used as an excitation source. The Raman characterization was carried out at room temperature. The morphology and interfacial microstructure of raw carbon fiber and C_f_ @PR composite powder were observed by scanning electron microscopy (SEM, SU9000, Hitachi, Japan). The microstructure and surface roughness of raw carbon fiber and C_f_ @PR composite powder were analyzed by atom force microscopy (AFM) (NTEGRA, NT-MDT, Moscow, Russia).

## 3. Results and Discussion

### 3.1. FTIR Spectra Analysis

**Figure 2:**
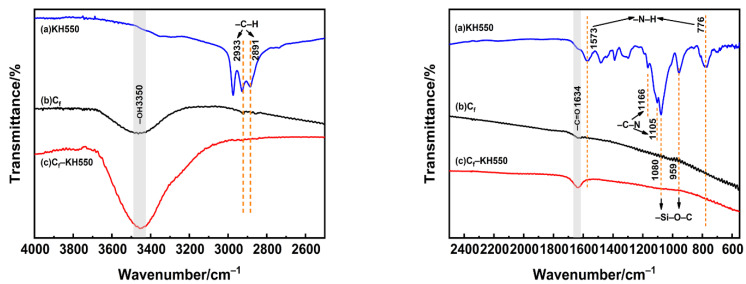
Fourier Transform Infrared Spectrometer (FTIR) spectra of the carbon fibers. (**a**) KH550, (**b**) before and, (**c**) after the modification of the KH550.

The FTIR spectra of KH550, raw carbon fiber, and the fiber modified by KH550 samples are shown in Figure 2 to analyze the surface functional groups. The curve a is for the KH550 including several peaks. The peaks at 1573 cm^−1^ and 776 cm^−1^ corresponded to the stretching vibration of –N–H [24]. The peaks at 1080 cm^−1^ and 959 cm^−1^ corresponded to the characteristic stretching vibration of –Si–O–C in KH550 [23]. The peaks at 1166 cm^−1^ and 1105 cm^−1^ corresponded to the stretching vibration of –C–N [25]. The peaks at 2933 cm^−1^ and 2891 cm^−1^ corresponded to the asymmetry and symmetry stretching vibration of –C–H, respectively [26].

For the carbon fiber before and after modification by KH550, the FTIR spectra included 3350 cm^−1^ and 1634 cm^−1^ two characteristic peaks. The 1634 cm^−1^ corresponded to the stretching vibration of –C=O, and the 3350 cm^−1^ corresponded to the stretching vibration of –O–H [27]. Meanwhile, compared with the raw carbon fiber, the intensity of the –OH peak increased significantly for the carbon fiber modified by KH550, which should be ascribed to the reversible hydrolysis equilibrium process of KH550 as shown in the following equation [28]. The occurrence of considerable –Si–OH and the formation of hydrogen bonds as well as the covalent bond between the –Si–OH and the carbon fiber led to the increment of the hydroxyl groups’ intensity for the modified carbon fiber [29]. Additionally, it should be noted that the FTIR spectra of carbon fiber after modification by KH550 did not express the characteristic peaks of KH550, which might be ascribed to the extremely low KH550 content and the limitation of FTIR detection sensitivity as well as the detection depth.

H_2_N(CH_2_)_3_Si(OC_2_H_5_)_3_ + H_2_O ⇌ H_2_N(CH_2_)_3_Si(OC_2_H_5_)_2_(OH) + CH_3_CH_2_OH

H_2_N(CH_2_)_3_Si(OC_2_H_5_)_2_(OH) + H_2_O ⇌ H_2_N(CH_2_)_3_Si(OC_2_H_5_)(OH)_2_ + CH_3_CH_2_OH

H_2_N(CH_2_)_3_Si(OC_2_H_5_)(OH)_2_ + H_2_O ⇌ H_2_N(CH_2_)_3_Si(OH)_3_+ CH_3_CH_2_OH

### 3.2. Raman Spectra Analysis

**Figure 3:**
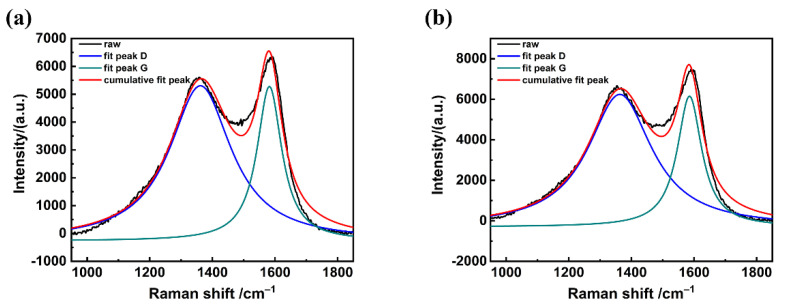
Raman spectra and fitting curves of the carbon fibers. (**a**) before and, (**b**) after the modification of the KH550.

Figure 3 shows the Raman spectra and fitting curves of the carbon fiber before and after the modification of KH550 to analyze the microcrystalline structure. The Raman spectra was composed of D-band characteristic bands ranged from 1330–1350 cm^−1^ and the G-band characteristic band ranged from 1580–1600 cm^−1^ [30]. The D-band represents the A_1g_ vibration mode of graphite crystallite, which indicates the disorder degree of the crystal structure [31]. The G-band represents the first-order scattering E_2g_ vibration mode of sp^2^ carbon atoms in the graphite structure, which indicates the integrity of the sp^2^ hybrid bonding structure [32]. The ratio of the D-band integral area and G-band integral area (R = I_D_/I_G_) indicates the disorder degree: the higher the R value, the more disorder degree would be expected [29].

Table 2 summarizes the Raman spectra calculating parameters including the D-band and G-band position, full width at half maxima (FWHM), and R value of the carbon fiber before and after the modification of KH550. Compared with the raw carbon fiber, the intensity of the G-band increased, indicating that the grafted KH550 changed the crystallite size and crystal edges. Meanwhile, it should be noted that the R value of the carbon-fiber- grafted KH550 increased from 2.49 to 2.64 according to the Tuinstra–Koenig equation as follows [33]

$$\mathrm{La}=\frac{\left(2.4\times 10^{-10}\right)\times \lambda^{4}}{R}$$

where La and λ represent the crystallite size and Raman laser wavelength. In our study, λ is 532 nm.

The increase of the R value corresponded to the decrease of La, which indicates that the introduction of KH550 increased the number of unsaturated carbon atoms at the corner and edges of the carbon fiber surface [34,35]. Therefore, the introduction of KH550 would increase the disorder degree.

### 3.3. Surface Elemental Composition Analysis

**Figure 4:**
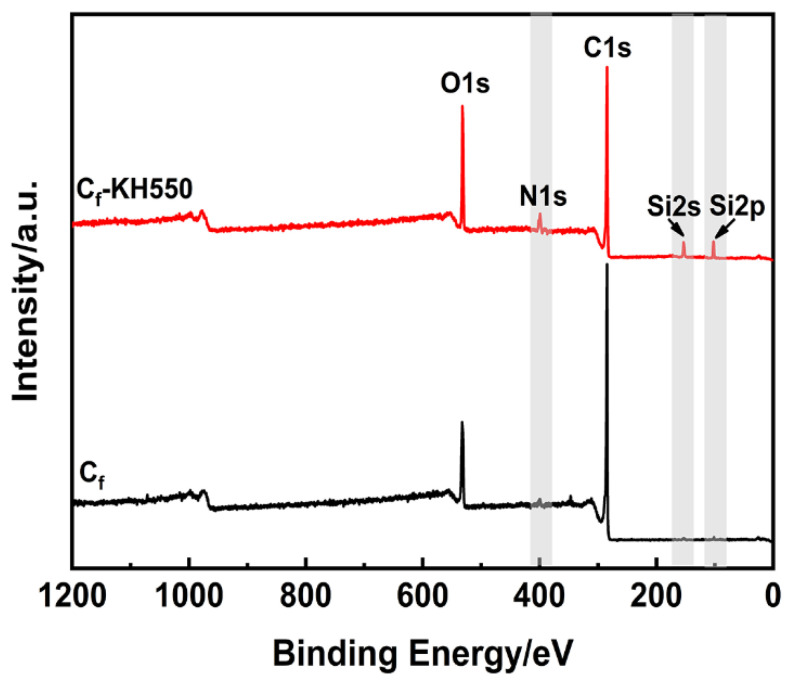
The XPS survey spectra of the carbon fiber before and after modification.

XPS analysis was used to analyze the surface elemental composition and concentration of the carbon fiber before and after modification of KH550 as shown in Figure 4. The XPS survey spectra indicated that the C_f_ and C_f_-KH550 samples were composed of four elements, including carbon (C), oxygen (O), silicon (Si), and nitrogen (O). Meanwhile, the intensity of the N and Si peaks were trivial for the raw carbon fiber compared with the carbon fiber modified by KH550. The presence of N and Si in the raw carbon fiber was due to the physical absorption of water, gas, and other substances.

Table 3 shows the surface elemental concentration of carbon fiber before and after the modification of KH550 corresponding to Figure 4. The surface of the raw carbon fiber was mainly composed of carbon (81.49%) and oxygen (15.78%). The relatively high oxygen content of unmodified carbon fiber provided considerable grafting reaction sites for KH550. After the surface modification of KH550, the relative contents of N, O, and Si increased from 2.04%, 15.78%, and 0.7% to 5.41%, 19.01%, and 5.66%, respectively. Among them, the obvious changes of Si and N content implied that KH550 was successfully introduced onto the surface of the carbon fiber. Correspondingly, the relative content of C predominant decreased from 81.49% to 69.92%, which resulted in an increase of the N/C, O/C, and Si/C ratios from 0.0250, 0.1936, and 0.0086 to 0.0774, 0.2719, and 0.0810, respectively.

**Figure 5:**
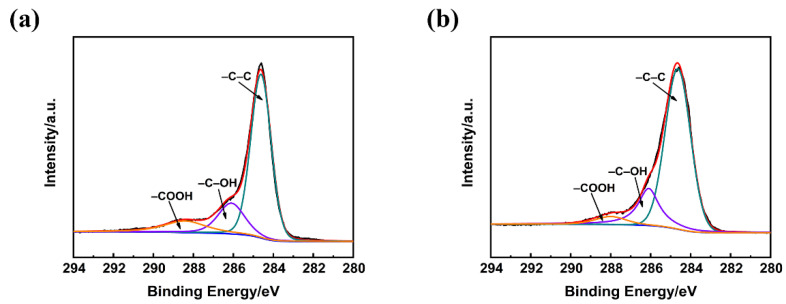
The C1s XPS spectra of (**a**) raw carbon fiber (**b**) carbon fiber modified by KH550.

The high resolution C1s XPS spectra was deconvoluted into three peaks including graphitic carbon (–C–C, 284.6 eV), hydroxyl (–C–OH, 286.3–286.5 eV), and carboxyl (–COOH, 287.6–288.3 eV) as shown in Figure 5 [36,37,38,39]. The –C–C band at 284.6 eV is mainly ascribed to the disorderly layered graphite stacking structure of carbon fiber [40]. The corresponding relative contents of the three bonds are shown in Table 4.

Compared with raw carbon fiber, the relative contents of –C–C and –C–OH on the surface of the carbon fiber modified by KH550 increased from 68.34% and 18.68% to 73.96% and 20.65%, respectively, while the content of –COOH decreased. During the surface modification process, the introduction of the KH550 silane coupling agent gradually hydrolyzed to generate considerable Si–OH bonds. Meanwhile, the adequately hydrolyzed KH550 reacted with the –OH on the fiber surface to form a chemical bond resulting in the increase of –OH and –C–C bonds, so that the number of –COOH bonds decreased. The deconvolution results of the C1s XPS spectra corresponded to the aforementioned FTIR results.

### 3.4. Surface Morphology of C_f_ @PR

**Figure 6:**
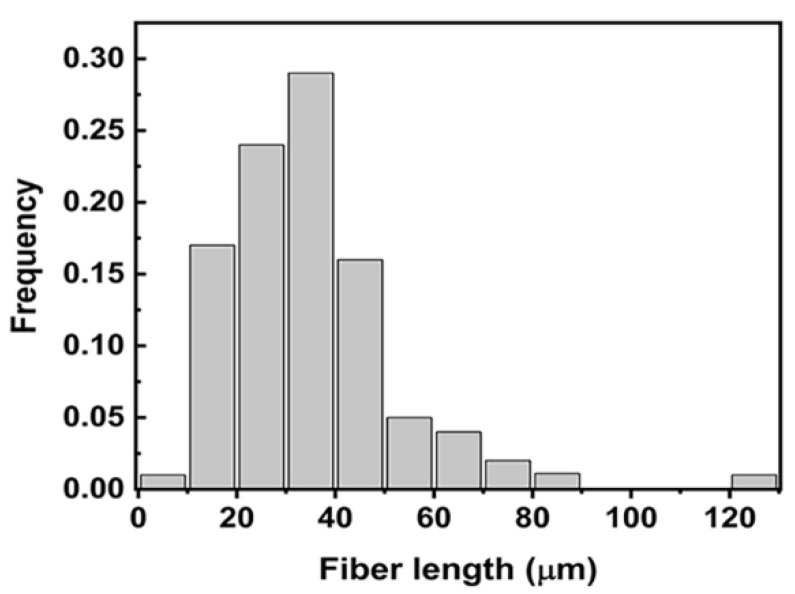
The length distribution of raw carbon fiber.

The fiber length distribution was calculated and is shown in Figure 6. The chopped C_f_ is composed of various lengths ranging from 1 μm to 130 μm, and the mean fiber length is 34.75 μm.

**Figure 7:**
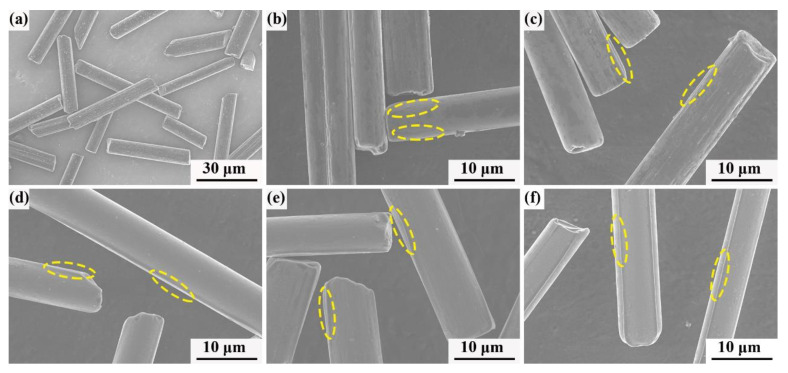
The SEM images of C_f_ @PR composite powders with different volumes of the PR binder. (**a**) 0 vol%, (**b**) 10 vol%, (**c**) 15 vol%, (**d**) 20 vol%, (**e**) 25 vol%, (**f**) 30 vol%.

Figure 7 depicts the SEM image of the C_f_ raw materials and the core-shell chopped C_f_ @phenolic resin composite with different PR contents. The raw chopped C_f_ had a rough surface with considerable grooves (Figure 7a). The formation of grooves was ascribed to the process of the raw filaments integrated into the tow [41]. Notably, the grooves can act as mechanical interlocking sites [22], which is a benefit of the formation of C_f_ @phenolic resin composite powder. Simultaneously, there existed many tiny fragments on the surface of the carbon fiber that were formed by the procedure of processing the continuous fibers into the chopped fibers.

Figure 7b–f show that the chopped C_f_ @PR composite powders have a PR volume content of 10 vol%, 15 vol%, 20 vol%, 25 vol%, 30 vol%, respectively. Due to the presence of the KH550 transition layer, the PR coating homogeneously dispersed on the surface of carbon fiber, hiding its original morphology and surface grooves. When the PR content is lower than 20 vol%, the interface between the carbon fiber and PR (as shown by the yellow circles) was blurred due to the low PR content, while when the PR content increased to 25 vol% and 30 vol%, the interface was distinct. Simultaneously, a few proportions of PR existed in the gap of fibers with increasing PR content. Furthermore, with the increment of PR content, the edges of the PR-coated carbon fibers were adhered to form a distinct crosslinked network structure ascribed to the decrease of the PR molecular chains’ distance during the drying process [42].

**Figure 8:**
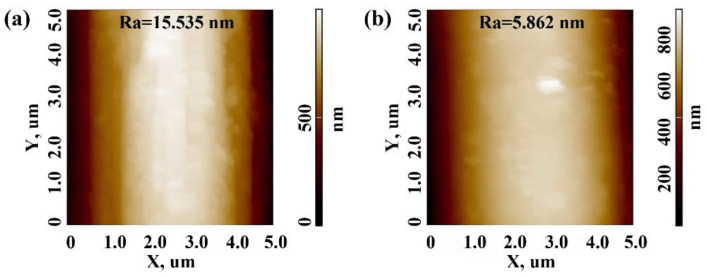
The atom force microscopy (AFM) image of (**a**) raw carbon fiber (**b**) chopped C_f_ @25 vol% phenolic resin.

The surface microstructure and roughness of raw carbon fiber and chopped C_f_ @25 vol% PR composite powder were further analyzed by AFM as shown in Figure 8. For the raw fiber, the calculating surface roughness value (Ra) was 15.535 nm. The presence of considerable longitudinal grooves resulted in a rough surface of raw fiber, whereas when the additional content of PR was 25 vol%, the roughness was significantly reduced (Ra = 5.862 nm). The decrement of roughness was ascribable to the PR coating on the fiber surface covering the grooves, leading to a relatively smooth surface. The AFM results were consistent with the aforementioned SEM results.

### 3.5. The Laser Absorption Analysis of C_f_ @PR

**Figure 9:**
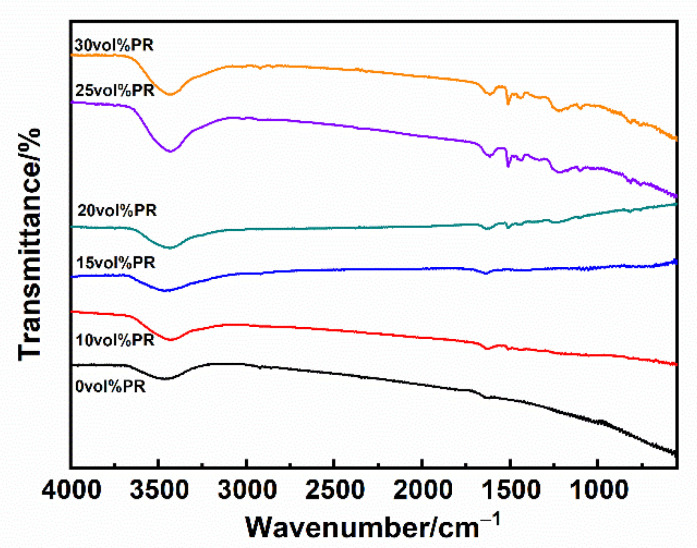
The laser absorption analysis of the C_f_ @PR composite powder with different PR contents.

For the indirect SLS process, the binder absorbs the energy coming from the laser beams to soften and bond the raw powders together. Therefore, the powders used for the SLS process should exhibit a relatively high laser absorption at a specific wavelength [43]. Normally for SLS, a CO_2_ laser with a wavelength of 10.6 μm is selected the energy source, and the polymer materials exhibit high laser absorption within the characteristic band ranging from 1300 cm^−1^ to 400 cm^−1^ (7.7~25 μm), normally [44]. Additionally, the mixed powder shows the hybrid absorption, which can be expressed by the follow equation [45]

(1) $$\alpha =\alpha_{b}\varphi_{b}+\;\alpha_{c}\varphi_{c}$$

where the *α*, *α_b_*, *α_c_*, *φ_b_*, and *φ_c_* represent the total absorption, the absorption of binder, the absorption of ceramic powder, the volume fraction of binder, and the volume fraction of ceramic powder, respectively. While we have fabricated the core-shell chopped C_f_ @phenolic resin composite powder, it shows a relative high laser absorption in this study. Figure 9 shows the laser absorption analysis of the C_f_ @PR composite powder with different PR content. As shown in Figure 9, with an increase of wavelength ranging from 1500 cm^−1^ to 550 cm^−1^(6.67~18.18 μm), the C_f_ @PR composite powder showed increased intensity, which indicates a decrease of transmittance and an increase of absorption. Further, with an increase of PR content, the C_f_ @PR composite powder showed intensive absorption compared with the C_f_ @PR composite powder without the addition of PR. This suggested that the addition of PR was beneficial to the improvement of the C_f_ @PR composite powder. Meanwhile, when the PR content increased to 30 vol%, the laser absorption did not increase significantly, which might be ascribed to the limitation of laser penetration depth. The laser-matter interaction is essentially an energy dissipation process, including deflection, absorption, and scatter (primary dissipation), heat equilibration (secondary dissipation), and long-range heat conduction (tertiary dissipation) [46], which results in the formation of a hierarchical heterogeneous structure due to the limitation of laser penetration depth and heat conduction as well as radiation [47]. Therefore, when the PR content increased to 25 vol% and 30 vol%, the PR coating had reached a certain thickness; it might be difficult to penetrate the thick PR coating [48], and thus the laser absorption did not increase obviously. Thus, the optimal PR content was 25 vol%.

## 4. Conclusions

A core-shell chopped C_f_ @phenolic resin composite powder with excellent laser absorption ratio for laser additive manufacturing was fabricated by the surface modification of a silane coupling agent combined with ball milling. The effect of the KH550 silane coupling agent and phenolic resin on the surface elemental composition, morphology, and laser absorption ratio of C_f_ @phenolic resin composite powder was investigated. We can draw the following conclusions:

(1) The FTIR, Raman, and XPS indicated that the KH550 has been grafted onto the surface of carbon surface successfully. After the surface modification, the concentration of the Si and N elements on the surface of the fiber changed significantly.
(2) The introduction of KH550 improves the wettability and compatibility between the carbon fiber and phenolic resin, which resulted in a homogeneous PR coating on the surface of carbon fiber. When the content of PR was higher than 20 vol%, an interface between the fiber and the PR was observed.
(3) The C_f_ @PR composite powder exhibited excellent laser absorption, ranging from 1500 cm^−1^ to 550 cm^−1^ (6.67~18.18 μm), which may be applicable for a CO_2_ laser during laser additive manufacturing. The optimal PR content was 25 vol%.

## Figures and Tables

**Table 1:** Basic physical properties of carbon fiber.

Item	Specification
Density	1.76 g/cm^3^
Monofilament diameter	6–8 μm
Oxygen content	0.58 wt%

**Table 2:** The Raman spectra calculating parameters of raw and KH550-modified carbon fiber.

Samples	D-Band		G-Band		R = I_D_/I_G_
	Position/cm^−1^	FWHM/cm^−1^	Position/cm^−1^	FWHM/cm^−1^	
C_f_	1361.81	239.23	1582.00	96.37	2.49
C_f_-KH550	1363.11	247.75	1585.52	95.32	2.64

**Table 3:** The surface elemental composition and concentration of carbon fiber before and after modification from the XPS survey.

Samples	Peak Designation	Band (eV)	At% Conc.	Atomic Ratio		
				N/C	O/C	Si/C
**C_f_**	C_1s_	284.64	81.49	0.0250	0.1936	0.0086
	N_1s_	400.36	2.04			
	O_1s_	532.17	15.78			
	Si_2p_	101.94	0.7			
C_f_-KH550	C_1s_	284.63	69.92	0.0774	0.2719	0.0810
	N_1s_	399.5	5.41			
	O_1s_	532.16	19.01			
	Si_2p_	102.48	5.66			

**Table 4:** The contents of surface functional groups from the deconvolution of C1s XPS spectra.

Sample	–C–C	–C–OH	–COOH
C_f_	68.34%	18.68%	12.98%
C_f_-KH550	73.96%	20.65%	5.39%

## Data Availability

The data presented in this study are available on request from the corresponding author.
